# Targeting E3 ubiquitin ligases: a new frontier in idiopathic pulmonary fibrosis treatment

**DOI:** 10.3389/fimmu.2025.1618424

**Published:** 2025-08-18

**Authors:** Kun Zhang, Hui Yuan, Lin Shi

**Affiliations:** Department of Pediatric Cardiology, Shandong Provincial Hospital Affiliated to Shandong First Medical University, Jinan, China

**Keywords:** idiopathic pulmonary fibrosis, E3 ubiquitin ligase, transforming growth factor-β, epithelial-mesenchymal transition, profibrotic fibroblast

## Abstract

Ubiquitination is a modification prevalent in eukaryotic cells. Disruptions in ubiquitination processes can have detrimental effects, potentially leading to diseases that endanger life. E3 ubiquitin ligases specifically recognize substrate proteins during ubiquitin modification, regulating intracellular protein levels and functions through the ubiquitin-proteasome pathway or TGF-β signal transduction. In recent years, substantial evidence has emerged, emphasizing the pivotal role that E3 ubiquitin ligases play in the development of pulmonary fibrosis. Advancing our understanding of how E3 ubiquitin ligases interact with pulmonary fibrosis could reveal new therapeutic targets and treatments for idiopathic pulmonary fibrosis (IPF), as well as innovative approaches in diagnosis and therapy. This review explores known regulatory mechanisms and identifies E3 ligases that have been implicated in IPF development.

## 1 Introduction

**Figure 1:**
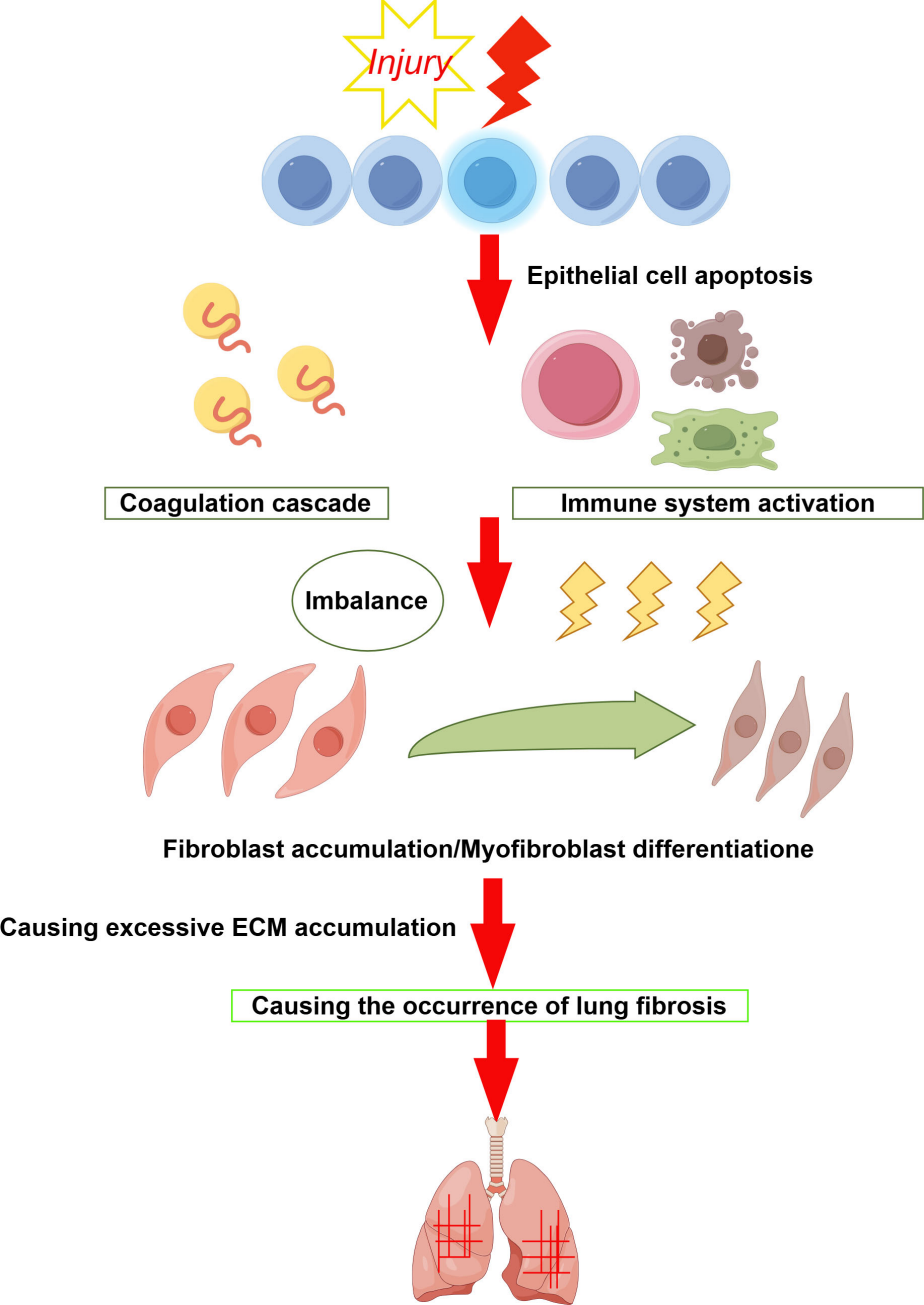
When the alveolar epithelium is damaged it triggers an immune response, a coagulation response, etc. Which leads to an imbalance between fibrosis and anti-fibrosis. This leads to an increase in fibroblasts and their transformation into myofibroblasts, which secrete excess extracellular matrix, ultimately leading to the development of pulmonary fibrosis.

IPF is a chronic, progressive, fibrotic form of interstitial pneumonia (1) and is the predominant form of idiopathic interstitial pneumonias (IIPs) (2). The pathogenesis of IPF remains elusive, primarily affecting older adults and confined to the lungs (3). According to relevant IPF studies, Idiopathic pulmonary fibrosis characterised by dyspnoea and progressive deterioration of lung function (4). Epidemiological studies indicate IPF affects approximately 3 million people globally, with an incidence of 2–30 cases per 100,000 person-years and a prevalence of 10–60 cases per 100,000 individuals (5, 6). Studies indicate that the global incidence and prevalence of IPF are increasing, the disease continues to significantly affect patients’ quality of life, leading to high rates of progressive respiratory failure and death (7). Males are more frequently affected than females, and the incidence increases with age (8). Currently, no curative treatments exist for IPF, and lung transplantation remains the most effective method for extending survival (9). Without transplantation, patients have a median survival of 3–5 years from diagnosis (10). Traditional treatments have included glucocorticoids, anti-inflammatory, and immunosuppressive drugs (11); however, studies have shown that glucocorticoids do not improve lung function or survival rates and are therefore not recommended for IPF patients (12, 13). Various factors, including smoking and exposure to metal and wood dust, have been identified as significant environmental contributors to IPF (14). Genetic predisposition plays a role in approximately 0.37% of cases (15, 16), often leading to overlooked familial fibrosis. Recent findings suggest mutations in the telomerase complex (17), activator A and C genes (18), and single nucleotide polymorphisms in the MUC5B promoter (19) may underpin some IPF cases. It is well-established that IPF involves the activation of epithelial cells (ECs) and age-related changes (20), with the fibrotic response resulting from aberrant activation of alveolar epithelial cells (AECs). These cells produce mediators that disrupt regeneration and promote the proliferation of lung-resident mesenchymal stem cells (MSCs), attract circulating MSCs, and stimulate epithelial-to-mesenchymal transition, ultimately leading to fibroblast and myofibroblast foci formation (21). These foci secrete excessive extracellular matrix, predominantly collagen, which results in scarring and lung destruction (22, 23). Diagnosing IPF is challenging and requires a multidisciplinary team including pulmonologists, radiologists, and pathologists (24). The diagnosis is typically confirmed through high-resolution CT scans showing typical interstitial pneumonia or pulmonary interstitial pneumonia, and occasionally surgical biopsy (25–28). A comprehensive medical history covering occupational and lifestyle exposures, immune markers, and a complete medication profile is crucial for differential diagnosis (29, 30). To enhance IPF diagnosis, studies have integrated circulating molecular markers such as MMPs, MMP-degrading proteins, pulmonary surfactant-associated protein D, osteopontin, and MMP7 (31). Research on the expression of lung gene products and inflammatory proteins continues to advance as potential diagnostic markers (32). There are studies that show that NLR (neutrophil to lymphocyte ratio) could be a more accurate predictor of inflammation than leukocyte counts. Therefore, the broad application of NLR levels in the diagnosis and prognosis of inflammatory diseases such as IPF (33). Emerging biomarkers, including epigenetic modifications, exosomes, and microbiome changes, have brought new diagnostic and monitoring tools for IPF (34). Further exploration of new molecular biomarkers and their mechanisms is necessary for early diagnosis and intervention, potentially delaying or halting disease progression in its early stages. **Figure 1** depicts the pathogenesis of idiopathic pulmonary fibrosis (IPF).

**Figure 2:**
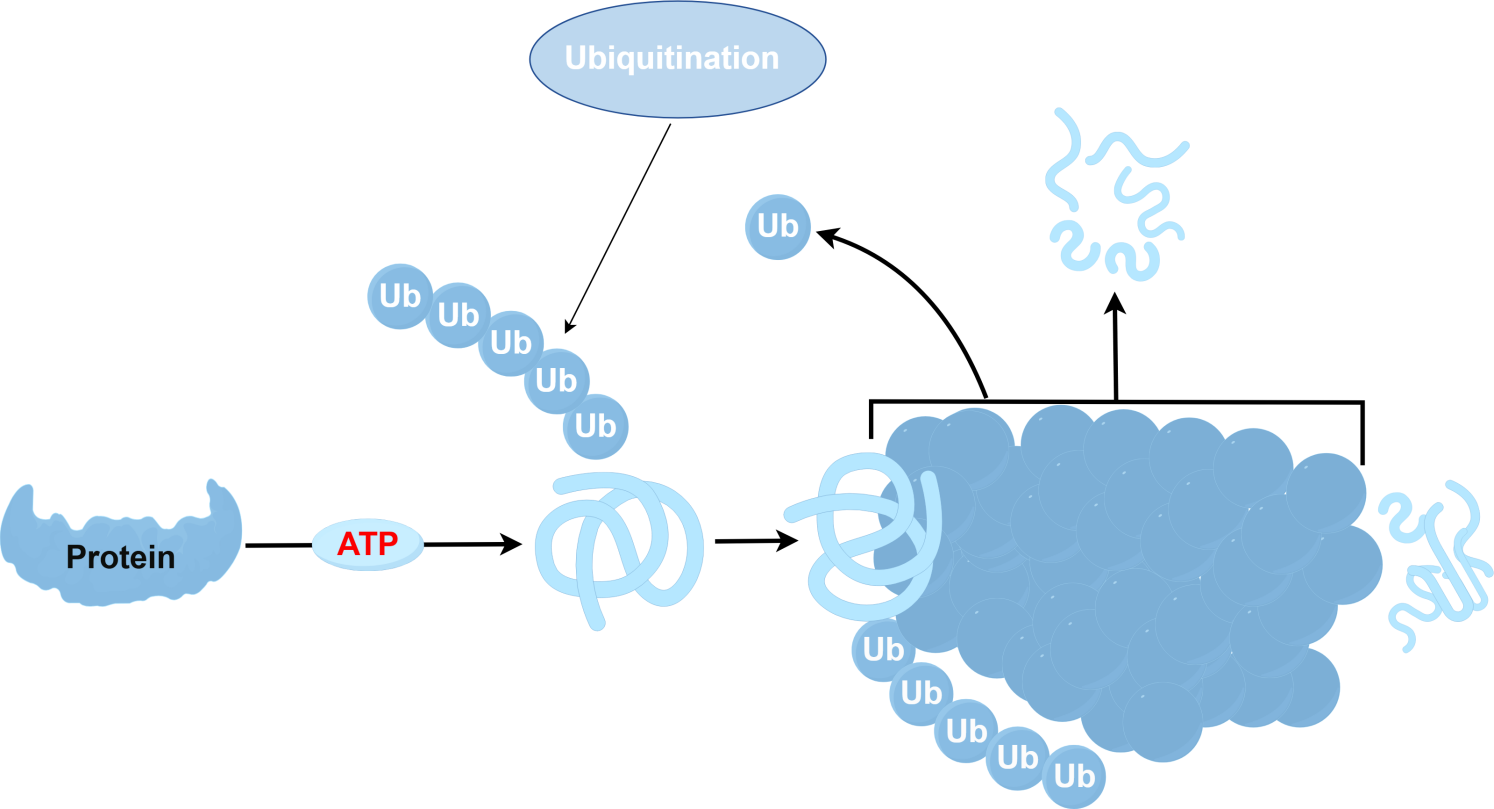
Schematic diagram of the ubiquitination process.

The ubiquitination process involves a series of enzymes: the ubiquitin-activating enzyme (E1), ubiquitin-conjugating enzyme (E2), and ubiquitin ligase (E3) (35–37). Among these, the E3 ubiquitin ligase stands out as an essential part of this pathway (38), tasked with recognizing specific target proteins (39). There exist two major families of E3 ligases within eukaryotes: the HECT and the RING domain families (40). Additionally, research has recently brought to light a further class known as the U-box protein family, characterized by a domain akin to the RING-finger (41). All E3 ligases have the unique function of linking a specific E2 to a target protein, facilitating the transfer of ubiquitin from E2 enzymes to their substrates, sometimes via a covalent E3 ubiquitin thioester intermediate (42). The specificity of ubiquitination hinges on the E2-E3-substrate interactions. The function of the E3 ligase is predominantly governed by the RING domain, which binds a thioester intermediate from E2 to facilitate ubiquitin release (43). Ubiquitin E3 ligases are pivotal in cell signaling, and the diversity within the E3 domain family has increasingly garnered attention. Nonetheless, the understanding of their biological functions, physiological partners, modes of action, and mechanisms remains nascent. Studies have shown that E2-E3 complexes can ubiquitinate substrate lysines and synthesize ubiquitin chains of various lysines (44), affecting substrates by regulating their structure, function, assembly, location, and proteasome-dependent degradation (45). While non-lysine ubiquitination has been observed, the corresponding E3 ligase remains unidentified. Previous research indicates that the ubiquitin-proteasome systems (UPS) and lysosomal degradation pathways, including autophagy, predominantly govern protein turnover. The ubiquitination process is shown in **Figure 2** (46, 47). However, recent research indicates that HECT-RING-finger and U-box-type E3 ubiquitin ligases might promote the advancement of pulmonary fibrosis by influencing the TGF-β-Smad pathway, which mediates epithelial mesenchymal transition (EMT) (48, 49). As a result, not only do fibroblasts evolve into myofibroblasts, contributing to the deposition of extracellular matrix and serving as crucial effector cells in IPF progression (50), but endothelial cells are also implicated significantly (51). Consequently, the interactions between the EMT and ubiquitin ligase signaling pathways are critical in fostering the development of IPF.

## 2 Main content

### 2.1 EMT and IPF

EMT represents a pathophysiological process wherein ECs lose their characteristic features and take on mesenchymal properties (52). This transition typically occurs under three conditions: development, cancer, and fibrosis, all of which involve tissue damage and remodeling that disrupt standard tissue homeostasis. During EMT, the loss of epithelial markers like E-cadherin and cytokeratin occurs alongside changes in surfactant production, and the adoption of mesenchymal markers (53) such as N-cadherin and fibronectin. This change leads to a rise in the secretion of matrix components or metalloproteinases, governed by various extracellular ligands. Myofibroblasts, pivotal effector cells in fibrosis, express alpha-smooth muscle actin (α-SMA) and proliferate following lung injury, which boosts collagen synthesis and fibrosis development. The precise origin of myofibroblasts in pulmonary fibrosis remains a subject for ongoing research.

### 2.2 Endothelial cells and IPF

Recent studies suggest that potential sources of myofibroblasts include local fibroblasts, circulating fibroblasts, tissue-resident pulmonary mesenchymal cells, and progenitor stem cells from epidermal and bone marrow sources (54). Notably, endothelial cells can also transform into myofibroblasts (55), a process known as EMT. During this transition, endothelial cells downregulate markers such as CD31 and vascular endothelial adhesion molecules, while expressing mesenchymal proteins like vimentin, α-SMA, and type I collagen. This transformation contributes to fibrosis pathogenesis through excessive collagen secretion and deposition, allowing these cells to migrate into surrounding tissues. High concentrations of α-SMA and myofibroblasts, transformed from endothelial cells, are observed in fibrotic lesions. It is hypothesized that α-SMA expression in microvessel endothelial cells is induced by combined activation of Ras and TGF-β.

### 2.3 TGF-β in IPF

TGF-β, a dimeric polypeptide growth factor (56, 57), is a key regulator of EMT, particularly noted for altering the phenotype of normal fibroblasts during tissue repair. TGF-β plays a crucial role in orchestrating the recruitment, activation, and differentiation of myofibroblasts during wound healing, initiated through the activation and release of TGF-β from various cells such as AECs, regulatory T cells (Treg), and activated platelets (58). As a significant pro-fibrotic mediator, TGF-β also mobilizes and activates monocytes and fibroblasts during fibrosis, facilitating fibroblast differentiation, inflammatory cell recruitment, and augmented extracellular matrix production and deposition (59–61). Acting as an immunosuppressive cytokine, TGF-β regulates cell proliferation, differentiation, immunomodulation, embryonic development, and angiogenesis (62) through diverse mechanisms. It stimulates gene transcription and produces collagen, fibronectin, and proteoglycans, enhancing extracellular matrix output and deposition (63). The TGF-β signaling system, with its complexity and interactions with other cell signaling pathways via TGF-β receptors (TβRs) (64), demands further clarification of its actions and mechanisms to aid in developing effective clinical treatments for IPF.

### 2.4 TGF-β signal pathway in IPF

TGF-β signaling is pivotal in fibrogenesis, facilitating the upregulation of fibrosis-associated genes and driving EMT, which contributes to fibrosis in the lungs and kidneys (65). It activates receptor complexes TβRI and TβRII, initiating a Smad-dependent cascade that enhances Smad2/3 activation (66). These activated proteins interact with co-chaperones Smads and Smad4, migrating into the nucleus to initiate transcription of target genes like collagen and fibronectin. Beyond the Smad pathways, TGF-β also influences tissue fibrosis through non-Smad mechanisms such as the Ras-MAPK, PI3K-Akt, and Par6-Smurf1 pathways, while concurrently inhibiting the TGF-β-Smad pathway by encouraging I-Smad binding to active TβRI, thus hindering R-Smad phosphorylation (67–69). E3 ubiquitin ligases play a role in regulating EMT and pulmonary fibrosis by targeting proteins for ubiquitination through the ubiquitin-proteasome pathway or by modulating TGF-β signaling. Notably, specific E3 ubiquitin ligases have been implicated in pulmonary fibrosis. This review summarizes the E3 ligases associated with IPF in recent years.

### 2.5 E3 ubiquitin ligase promoting pulmonary fibrosis

Here, we discuss our current understanding of E3 ubiquitin ligases thatexecutes its effector functions in IPF (**Table 1**). Smurfs(Smad-ubiquitin regulatory factors) are part of the HECT-type E3 ligases (82). Among the 30 HECT E3 ligases identified in mammals (83), only a few, such as Smurf1/2, have well-documented functions. For instance, Smurf1 and Smurf2, which facilitate the ubiquitin-dependent degradation of the unmethylated Smad7-TβR complex, reduce TGF-βsignaling and may thus mitigate pulmonary fibrosis. Despite observations of increased Smurf1 expression in conditions like sarcoidosis and IPF, suggesting its involvement in IPF pathogenesis (84), further investigation is necessary to confirm this relationship. Moreover, Smurf2 acts as a negative regulator of TGF-β signaling (85) by binding directly with Smad2 and Smad3 via a linker region featuring the PY motif. This interaction leads to the proteasomal degradation of Smad2 and Smad3 (86). Furthermore, complexes formed between Smurf2 and Smad7 target the activated TGF-β type I receptor for ubiquitination and degradation (85). *In vitro* studies from Smurf2 knockout mice show that deletion of Smurf2 increases smad3 activity and inhibits smad3 complex formation by inducing multiple monoubiquitination of smad3. In addition, miR-424-regulated Smurf2 influences myofibroblast differentiation during EMT, contributing to pulmonary and obstructive renal fibrosis. miR411-3p and miR-27a-3p are known to mitigate bleomycin-induced pulmonary fibrosis by downregulating Smurf2 (70).

**Table 1:** Role of E3 ubiquitin ligases in idiopathic pulmonary fibrosis.

E3 ubiquitin ligases	Models	Promot or inhibit	Mechanisms	Ref no.
Smurf1/2/3/7	IPF	promot	TGF-β signaling	(70)
Skp2	BLM-induced IPF	promot	P27 ubiquitination and degradation	(71)
FIEL1	IPF	promot	TGF-β signaling	(72)
BARD1	IPF	promot	Apoptosis and influences cell proliferation	(73)
TRIM2	BLM-induced IPF	promot	Dle2/miR-369-3p/TRIM2 axis	(74)
Arkadia	IPF	promot	TGF-β-Smad pathway	(75)
LOMP	IPF	promot	TGF-β-Smad pathway	(76)
NEDD4L	LFs	inhibit	Wnt/β-catenin signaling	(77)
NEDD4-2	IPF	inhibit	TGF-β signaling	(78)
STUB1	IPF	inhibit	TGF-β signaling	(79)
Fbxw7	BLM-induced IPF	inhibit	miR-155/Fbxw7/TGF-βaxis	(80)
TIF1γ	IPF	inhibit	HSPB5/TIF1γ/Smad4pathway	(81)

Skp2, part of the SCF-Skp2 ubiquitin ligase complex, also plays a role in increasing mesenchymal fibroblasts in response to bleomycin (87–89), affecting pulmonary fibrosis progression. This complex is crucial for the proteasome-dependent degradation of various growth inhibitors, including CDK inhibitors (e.g., p27, p21, p57) and tumor suppressor proteins (e.g., p130 and Tob1) (90), by facilitating their ubiquitination. In the BLM-mediated fibrosis model, Skp2-deficient mice promote mesenchymal fibroblast proliferation and EMT, during the advancement of pulmonary fibrosis by mediating P27 degradation (71). While Skp2 is known to regulate cellular processes like apoptosis and fibrosis, its specific mechanisms require further exploration (90). Skp2 deficiency may reduce early inflammation, while Skp2 inhibitors suppress both inflammation and fibrosis by inhibiting lung cell proliferation and decreasing mesenchymal fibroblasts. This suggests that Skp2 inhibitors could be potential treatments for IPF (91). Previous studies have demonstrated that reducing Skp2 expression enhances the expression of inhibitors like p27 and p21 in pathogenic autoreactive Treg and increases Foxp3, a key transcription factor for Treg (92). This modulation limits effector T cell proliferation and fosters the conversion of pathogenic Treg to regulatory types. Conversely, Skp2 overexpression in regulatory Treg diminishes Foxp3 levels and impairs their function (93). Additionally, a deficiency in Skp2 may inhibit apoptosis in renal epithelial and stromal cells, potentially halting the progression of kidney fibrosis.

Fibrosis-induced E3 ligase 1 (FIEL1), a novel protein isomer encoded by KIAA0317, is a member of the RING domain E3 ubiquitin ligase family (94). Research has shown that FIEL1 modulates TGF-β signaling by targeting PIAS4 for ubiquitin-mediated degradation, thereby influencing the initiation and progression of fibrosis (72). FIEL1 also regulates the level of PIAS4 protein in MRC5 (human fetal lung primary fibroblasts) by ubiquitinating K31 in PIAS4 and marks numerous other proteins for ubiquitination and subsequent degradation (94). The presence of FIEL1 may alter the inflammatory response and trigger fibrotic damage in the lungs. Consequently, down-regulation of FIEL1 *in vivo* can ameliorate BLM-induced lung injury. PIAS4 binding to FIEL1 occurs only when phosphorylated by PKCζ, while GSK3β phosphorylation is necessary for PIAS4 targeting. Ectopic expression of FIEL1 in MRC5 cells enhances TGF-β signaling and fibrosis by disrupting PIAS4. A naturally occurring amino acid mutation (P779L, rs371610162) in FIEL1 has been identified with a significant protective effect in IPF. The FIEL1 inhibitor BC-1485, which also binds to the substrate PIAS4 in the HECT domain region, shows potent antifibrotic properties by stabilizing PIAS4 and suppressing TGF-β signaling in both murine models of pulmonary fibrosis and human MRC5 cells (95, 96). Elevated levels of FIEL1 and reduced PIAS4 protein expression in patients with IPF suggest that FIEL1 may regulate SMAD signaling through PIAS4, promoting SMAD translocation and suppressing fibrotic gene expression (97, 98). Recent studies indicate that ECs and fibroblasts may be involved in fibrosis via the FIEL1-PIAS4 pathway, with ongoing research aimed at identifying which cell types predominantly affect IPF through this mechanism. Therefore, FIEL1 has physiological significance in TGF-β signaling and IPF, and the inhibitor BC-1485 could provide a new treatment strategy for IPF.

BARD1, a tumor suppressor in concert with the breast cancer susceptibility gene BRCA1, plays a significant role in BRCA1-driven tumor suppression (99). The BRCA1-BARD1 ubiquitin ligase complex targets various cell-cycle regulatory proteins, such as centrosomal protein gamma-tubulin and Aurora kinase, for ubiquitination and subsequent degradation (100). BARD1 also induces apoptosis and influences cell proliferation by stabilizing p53 through its interaction. Notably, BARD1 β variant, which is devoid of a RING structure yet contains an Aurora kinase binding domain, is vital for the proliferation of general cells and fibroblasts, suggesting its potential role in the advancement of pulmonary fibrosis (73). In cellular environments, BRCA1 predominantly forms a heterodimer with BARD1, co-localizing with DNA replication and repair machinery upon DNA damage, thereby playing a crucial role in DNA repair and cell cycle regulation (101–104). Additionally, BARD1’s expression may be upregulated by hypoxia and TGF-β, implicating a potential indirect influence on the TGF-β signaling pathway. Western blots and RT-PCR analyses have shown that E-cadherin expression remains unchanged in cells overexpressing BARD1β, suggesting that BARD1β may affect the accumulation and cytoplasmic localization of fibronectin in A549 cells (105). Mouse models of pulmonary fibrosis have shown upregulation of BARD1, similar to cells induced by BARD1, where expression of apoptosis markers p53 and Bax was detected in rat fibrotic lung tissue (106). Immunohistochemical results have confirmed that the localization of BARD1 expression is confined exclusively to fibrotic area. BARD1 could act as a downstream activator in EC apoptosis and fibroblast proliferation, making it a critical target for therapeutic intervention in pulmonary fibrosis treatment.

The Tripartite Motif (TRIM) family of E3 ubiquitin ligases, recognized for their conservation across species, regulates diverse biological functions including cell proliferation, apoptosis, differentiation, metastasis, gene transcription, signal transduction, inflammatory responses, and immune reactions (107). TRIM2, belonging to the RING domain-containing subfamily of E3 ligases, regulates vimentin ubiquitination in lung squamous cell carcinoma cells (108). Moreover, TRIM2 is implicated in enhancing the proliferation, migration, and invasion of colorectal cancer cells through EMT (109) and promotes osteosarcoma progression and metastasis via the PI3K/KPB signaling pathway. Interaction between Dle2 and TRIM2 with miR-369-3p, which TRIM2 regulates (74), was observed to increase in lung tissue from mice with BLM-induced fibrosis and in TGF-β1-stimulated A549 cells, where miR-369-3p levels were reduced. Consequently, Dle2 may regulate TRIM2 by upregulating miR-369-3p, influencing the occurrence and progression of EMT and IPF. The specific mechanisms by which TRIM2 affects IPF warrant further study. TRIM47 targets PPM1A for ubiquitination, leading to its degradation via the 26S proteasome complex, which in turn increases the phosphorylation of Smad2/3 and contributes to lung fibrosis (110). Immunoprecipitation studies showed TRIM47’s interaction with PPM1A in HELF cells, and the reduction in PPM1A protein levels caused by TRIM47 can be suppressed by the proteasome inhibitor MG132. TRIM47 reduces PPM1A protein levels but not its mRNA expression, primarily acting through post-translational modifications. Inhibiting TRIM47 expression can suppress the expression of Vimentin, α-SMA, and CTGF protein, reduce Smad2/3 phosphorylation and hydroxyproline production, thereby mitigating pulmonary fibrosis. Conversely, overexpression of TRIM47 reversed these effects. Melittin has been shown to downregulate TRIM47 expression, which may contribute to its inhibitory effects on HELF fibrosis (111). The exact interactions between melittin, TGF-β, Smad2/3, and TRIM47, and whether melittin directly targets TRIM47 or acts through other proteins, remain to be further elucidated.

Arkadia, a single RING-type E3 ubiquitin ligase, influences fibrosis by modulating the TGF-β-Smad pathway (75). It promotes the degradation of methylated inhibitory Smad7 by Set9, thus facilitating bleomycin-induced and Ad-TGF-β-induced pulmonary fibrosis (112). Similarly, the SUMO-E3 ligase plays a role in directing nuclear export of SMAD proteins, exacerbating pulmonary fibrosis by enhancing TGF-β activity. Arkadia also influences the transcription of pro-fibrogenic genes and their products, including collagen, smooth muscle activity, and fibronectin. Genetic alterations have also been linked to fibrosis (113). LIM domain-only protein 7 (LMO7), a member of the PDZ and LIM domain-containing protein family. A new study shows the importance of LMO7 in BLM induced pulmonary fibrosis. LMO7 was observed to promote profibrotic fibroblast polarization via TGF-β/SMAD signalling by mediating the degradation of SMAD7 (76).

### 2.6 E3 ubiquitin ligase inhibits pulmonary fibrosis

NEDD4L, an HECT-type E3 ligase anchored to the membrane, targets several membrane proteins, notably the epithelial sodium channel (ENaC) and TβR. Conditional deletion studies in mice suggest that overexpression of NEDD4L suppresses IPF formation and progression by attenuating proliferation, invasion, and differentiation of lung fibroblasts. The deletion of NEDD4L exacerbates IPF in murine models, underscoring its protective role in bleomycin-induced pulmonary fibrosis by mediating the degradation of ENaC and TβR (77). In type II lung ECs, specific deletion of NEDD4L elevates ENaC protein levels, resulting in a cystic fibrosis-like condition. Further studies indicate that NEDD4L may restrain IPF progression by promoting β-catenin ubiquitination, thereby inhibiting Wnt/β-catenin signaling and suppressing the CTHRC1/HIF-1α axis, which diminishes the activity and differentiation of lung fibroblasts (LFs).

Similar to NEDD4L, the membrane-associated E3 ubiquitin ligase NEDD4–2 regulates various proteins, including ENaC, proSP-C, and Smad2/3 (114). These proteins are critical in regulating TGF-β signaling and surfactant biogenesis for EC homeostasis and lung homeostasis. Deletion of Nedd4–2 leads to ubiquitination and phosphorylation of Smad2/3, resulting in dysregulation of TGF-β signaling and impaired mucociliary clearance, thereby affecting pulmonary fibrosis (115). Conditional knockout studies of nedd4–2 in mice have demonstrated decreased transcription of AT2 cell markers such as surfactant protein C (Sftpc) and surfactant protein D (Sftpd), alongside increased expression of pro-fibrotic markers such as vimentin (Vim) and fibronectin (Fn1). Administration of the clinical drug pirfenidone in these knockout mice has been shown to suppress activation of the PTEN pathway, inhibit fibroblast proliferation, and reduce TGF-β-induced phosphorylation of Smad2/3 as well as the expression of α-SMA and collagen (78). These findings suggest that pirfenidone acts on the TGF-β pathway to mitigate the development and severity of pulmonary fibrosis. Mice born without Nedd4–2 in their lung epithelium succumb shortly after birth due to alveolar inflammation and fibrosis. Similarly, in neonatal mice, deletion of Nedd4–2 promotes damage to alveolar type 2 (AT2) cells, impaired mucociliary clearance, and increased ENaC activity (116). Interestingly, in congenitally Nedd4–2 deficient mice, we observed an age-dependent variation in the expression of lung mucin proteins Muc5b and Muc5ac, leading to more severe bleomycin-induced pulmonary fibrosis. This may be attributed to the increased sensitivity of the neonatal lung and its developmental stage. The established model in juveniles is essential for investigating the pathogenesis of Neonatal IPF *in vivo* and identifying potential biomarkers and therapeutic targets.

The PTEN-induced kinase 1 (PINK1)/PARK2 pathway facilitates selective mitochondrial autophagy, commonly referred to as mitochondrial autophagy (117). Mutations in PINK1 and PARK2 are associated with an accumulation of damaged mitochondria and Parkinson’s disease (118). Moreover, both PINK1 and PARK2 contribute to the regulation of mitochondrial autophagy, control of mitochondrial ROS (119), and modulation of lung EC death and senescence, playing crucial roles in the pathogenesis of IPF and chronic obstructive pulmonary disease (COPD) (120). However, the impact of mitochondrial autophagy on fibroblast phenotypic changes during pulmonary fibrosis development remains unclear. In alveolar macrophages (AMs) of IPF patients, mitochondrial morphology and transcription are compromised (121–123), and key regulators of mitochondrial homeostasis—PINK1, PARK2, and NRF1—are notably reduced. PINK1 is crucial for maintaining mitochondrial integrity through pathways dependent on and independent of mitochondrial autophagy (124). Knockout of the PINK1 gene increases mtROS production, leading to mitochondrial fragmentation or autophagy and subsequent clearance. Although recent studies showed diminished PINK1 expression in IPF lungs (125), no significant differences were found in PINK1 levels between normal lung fibroblasts (LF) and IPF LF, suggesting a unique role for PINK1 in IPF progression. However, deletion of PINK1 also results in reduced activity of MRC complex 1 in the absence of mitochondrial autophagy, warranting further investigation. Reduced PARK2 expression has been linked to impaired autophagy and accelerated EC senescence in COPD (126). PARK2 knockdown inhibits mitochondrial autophagy, activating the PDGFR/PI3K/AKT pathway, which promotes myofibroblast differentiation and proliferation. However, treatments with both an antioxidant and the PDGFR inhibitor AG1296 significantly curtailed these processes. PARK2 knockdown more effectively promotes myofibroblast differentiation than PINK1 knockdown. Moreover, in a PARK2 KO mouse model, both Masson trichrome staining and Sircol collagen detection demonstrated increased lung fibrosis development, with immunohistochemistry revealing an accumulation of p62 and ubiquitin-modified proteins. Treatment with AG1296 significantly mitigated pulmonary fibrosis in PARK2 KO mice, indicating that PARK2-mediated mitochondrial autophagy plays a crucial role in regulating the PDGFR signaling pathway and the pathogenesis of IPF (127). In contrast, no phenotypic changes were observed in fibroblasts from PINK1 KO mice, highlighting PARK2’s predominant role in IPF pathogenesis through its regulation of myofibroblast differentiation via mitochondrial autophagy (128). A study by Liguori et al. showed Pirfenidone (PFD) inhibits myofibroblast differentiation, induced by PARK2 knockdown, by decreasing mitochondrial ROS and influencing the PDGFR-PI3K-Akt signaling pathway. In PARK2 KO mice treated with bleomycin (BLM), Pirfenidone reversed enhanced lung fibrosis and oxidative alterations, verifying its anti-fibrotic effects and highlighting its potential as a therapeutic option for IPF (129).

U-box type E3 ligases, including STUB1 and CHIP, play roles in the ubiquitin-mediated degradation of Smads within the TGF-β pathway. Specifically, STUB1 (STIP1 homolog and U-box containing protein 1), an acknowledged E3 ubiquitin ligase for NOX4, promotes the degradation of Smad3 and NOX4, exerting a negative regulatory effect on the TGF-β pathway (79). Azithromycin (AZM) has been found to inhibit autophagy and contribute to the development of pulmonary fibrosis by increasing NOX4 ubiquitination through elevated STUB1 protein levels (130). AZM not only activates the proteasome but also boosts NOX4 degradation by specifically enhancing its ubiquitination via STUB1, which is crucial for mitigating TGF-β-mediated myofibroblast differentiation by regulating reactive oxygen species (ROS). Furthermore, research indicates that proteasome-dependent degradation of NOX4 is instrumental in the anti-fibrotic effects of Azithromycin (131). Consequently, STUB1-mediated degradation of NOX4 inhibits myofibroblast differentiation, playing a key role in reducing bleomycin-induced pulmonary fibrosis. Given Azithromycin’s dual function in suppressing autophagy and activating STUB1, it holds potential for therapeutic use in pulmonary fibrosis by targeting NOX4 and Smad3 degradation. In the context of COPD and allergic airway diseases triggered by allergens such as dust mites or egg proteins, TNF-related apoptosis-inducing ligand (TRAIL) can alleviate pulmonary fibrosis by up-regulating MID1 (E3 ubiquitin ligase), which inactivates protein phosphatase 2A (PP2A) (132). TRAIL promotes the interaction between the E3 ubiquitin ligase midline-1 (MID1) and the α4 regulatory subunit, thus inhibiting PP2A activation and reducing fibrosis progression. Elevated MID1 expression leads to decreased PP2A activity, resulting in enhanced phosphorylation of mitogen-activated protein kinases (MAPKs) and inhibitors of κBα (IκBα) proteins. This activation stimulates the p38 MAPK, c-Jun N-terminal kinase (JNK), and nuclear factor-κB (NF-κB) pathways. In models of bleomycin-induced pulmonary fibrosis, increased MID1 activity and decreased PP2A activity are observed. Conversely, the absence of TRAIL or the activation of PP2A can reverse these effects and enhance lung function, suggesting that the E3 ubiquitin ligase MID1 may serve as a viable target for IPF treatment.

The F-box and WD repeat domain-containing 7 (Fbxw7) protein, belonging to the F-box family, forms part of the SKP1-cullin-F-box-protein (SCF) ubiquitin ligase complex (133). Fbxw7 plays a key role as an inhibitor in the progression of pulmonary fibrosis (PF) in bleomycin-treated mice (80). Studies have shown a strong correlation between decreased expression of E3 ubiquitin ligase Fbxw7 in peripheral blood mononuclear cells from patients with IPF and increased disease severity. Additionally, the level of microRNA-155 (miR-155), which is associated with the degree of tissue damage in IPF, inversely affects Fbxw7 mRNA levels. In experiments involving myeloid cell-specific Fbxw7-deficient mice treated with bleomycin, an increase in TGF-β within pro-fibrotic macrophages was noted. Flow cytometry revealed heightened monocyte-macrophage accumulation in lung tissues and increased pro-inflammatory and pro-fibrotic cytokines such as TNF-α and IL-1β, leading to enhanced collagen deposition and exacerbation of bleomycin-induced pulmonary fibrosis. In models of LPS-induced lung injury, the absence of Fbxw7 in myeloid cells resulted in more severe injury and fibrosis compared to controls. Fbxw7 is also recognized as a tumor suppressor due to its ability to target oncogenic proteins like c-Myc, Notch, MCL1, and c-Jun. Co-immunoprecipitation assays demonstrate that Fbxw7 interacts with c-Jun, facilitating its K48-linked polyubiquitination and proteasomal degradation. The absence of Fbxw7 leads to elevated TGF-β expression in macrophages by diminishing c-Jun ubiquitination, thus intensifying pulmonary fibrosis (80). Additionally, Fbxw7 exhibits immunomodulatory effects that restrict effector T cell adaptability and endurance within the tumor microenvironment by inhibiting Notch signaling. Furthermore, Fbxw7 promotes antiviral innate immunity and may augment intestinal inflammation (80, 134), indicating its complex regulatory role in macrophage function across various tissue environments. Recent studies suggest that enhancer of zest homolog 2 (Ezh2) may increase H3K27me3 methylation at the Fbxw7 promoter, potentially repressing Fbxw7 expression, while IL-4 could reduce Fbxw7 activity to inhibit c-Jun degradation in fibrotic environments, thereby impacting pulmonary fibrosis. Contrarily, Fbw7 deficiency may prevent stress-induced senescence in type II AECs and reduce lung fibrosis in disease models. In Fbw7-deficient A549 cells, enhanced stability of TPP1 has been observed, which also contributes to the inhibition of pulmonary fibrosis. Consequently, Fbw7 inhibitors like TELEODin might represent promising therapeutic approaches for IPF management.

The αB-crystallin protein, or HSPB5, is part of the small heat shock proteins (sHSPs) family (135) and is involved in various types of fibrosis including renal, vascular, and pleuropulmonary. Triggered by TGF-β1, increased αB-crystallin levels impede the monoubiquitination of Smad4 by interfering with its E3 ubiquitin ligase, TIF1γ, thereby blocking Smad4’s nuclear export. This action effectively suppresses the fibrogenic activity of the TGF-β1-Smad4 pathway and restricts the proliferation and differentiation of myofibroblasts, resulting in significant fibrosis in mouse lungs. Moreover, TGF-β1 is known to promote EMT, and recent findings indicate that TIF1γ serves as a negative regulator of Smad4 in TGF-β1-driven EMT in mammary Ecs (81). In αB-crystallin deficient mice, the progression of fibrosis is mitigated by facilitating Smad4’s nuclear exit through the monoubiquitination of TIF1γ, thereby disrupting Smad4’s binding and blocking TGF-β1 signaling (136). HSP90 stabilizes the TGF-β receptor complex and aids the nuclear translocation of the Smad complex via its interaction with Smad4. Inhibitors of HSP90 enhance the stability of the TGF-β receptor and boost both typical and atypical TGF-β signaling pathways and EMT, subsequently repressing the expression of fibrosis-related genes (137–139). Thus, inhibitors of HSPB5 and HSP90 may represent promising therapeutic options for IPF.

**Figure 3:**
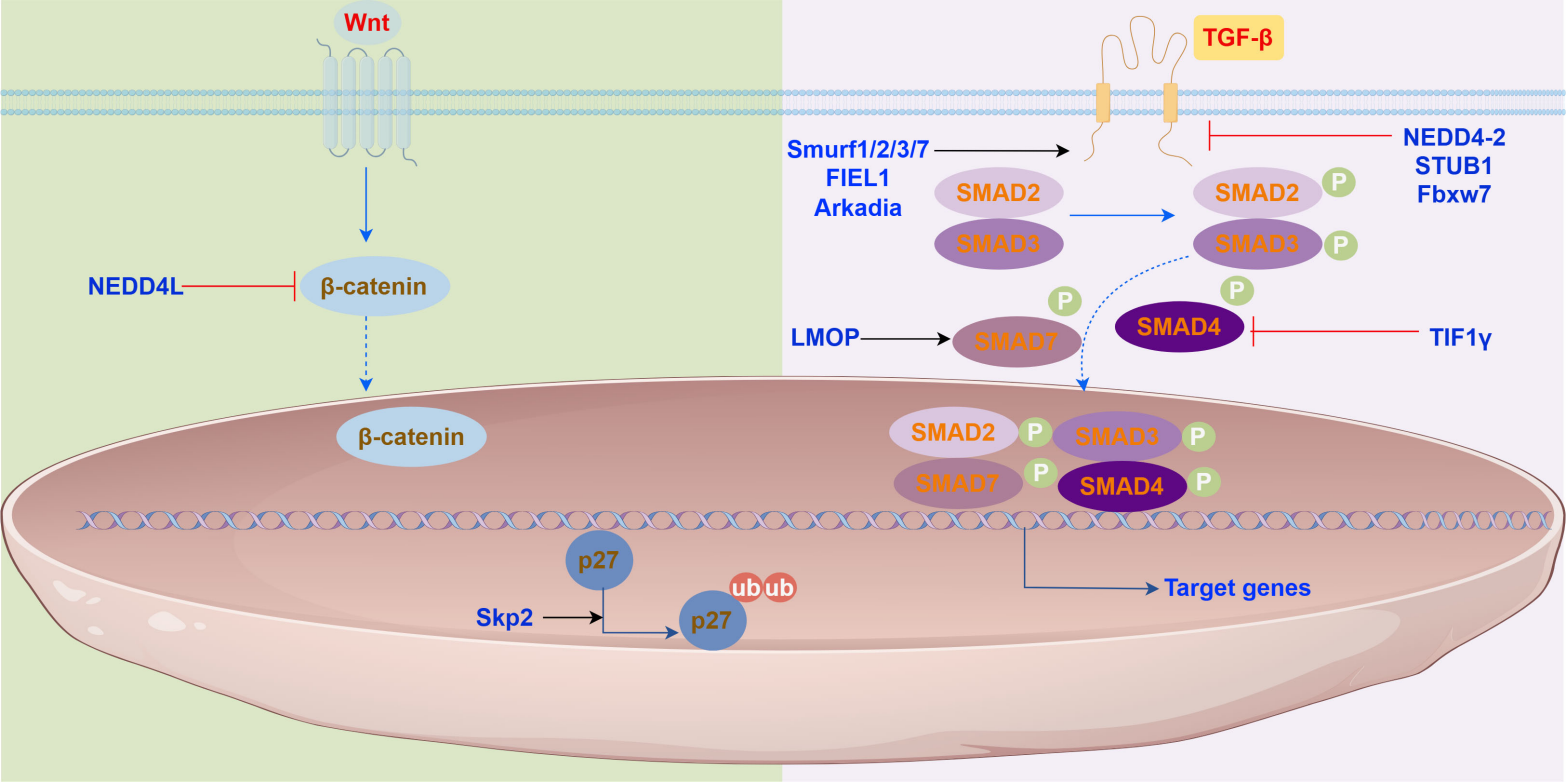
E3 ubiquitin ligases in the signaling pathways. E3 ubiquitin ligase play roles in (Left) the Wnt/β-catenin signaling pathway, (Right) the TGF-β/Smad signaling pathway.

Furthermore, Aging is the leading risk factor for IPF. Mitochondrial dysfunction accumulated during aging is a key link in the pathological process of IPF, and its core manifestations are energy metabolism disorders and increased oxidative stress. As a core performer of intracellular protein degradation and quality control, especially its role in maintaining mitochondrial health and antioxidant defense and regulating key signaling pathways, E3 ubiquitin ligase is a key molecular hub connecting aging, mitochondrial dysfunction and IPF pathogenesis. Understanding the expression, activity, and functional changes of specific E3 ligases such as Parkin in IPF lung tissue (140), and how they affect mitochondrial function and cell fate, is of great significance for unraveling the pathogenesis of IPF and developing new targeted therapeutic strategies. The effects and mechanisms of E3 ubiquitin ligases in IPF are shown in **Figure 3**.

## 3 Conclusions and perspectives

A variety of pathogenic factors lead to lung injury, which then triggers a cascade of events that progresses to pulmonary fibrosis. This process involves a complex architecture, and the cytokines and signaling pathways involved in signal transduction remain elusive. Although recent studies have highlighted the role of E3 ligases in the development of TGF-β signaling, their involvement in IPF is less documented. Moreover, the discovery of antifibrotic drugs such as AZM, BC450, and PFD, along with the clinical use of pirfenidone and nintedanib, suggests that targeting specific E3 ligases and their downstream effectors could transform IPF management. Furthermore, E3 ubiquitin ligase also plays an important role in various cancers (141), bony metastases are a common finding in numerous cancers, particularly breast, prostate and lung (142). In the future treatment disease will shift from prolonging survival to functional recovery and even reversal of some diseases with time. Therefore, it will be interesting to further explore the role of E3 ubiquitin ligase in bone metastasis. Despite the widespread use of various potential treatments in clinical trials, the median survival time for pulmonary fibrosis remains limited, the standard of care for IPF consisted of treatment with corticosteroids, azathioprine and other immunosuppressive medications, which have been shown to be associated with higher mortality (7). Lung transplantation is currently the only definitive treatment for improving the life quality of those with IPF, highlighting a significant unmet need for the advancement of antifibrotic therapies. Ubiquitination modifications, which involves targeting substrate molecules degradation, may be milder than directly inhibiting the activation of SMADs and has little effect on the basic activation of TGF-β signalling. Thus, the regulatory effect of E3 ubiquitin ligases on the TGF-β pathway may provide a new direction for the development of antifibrotic drugs. Meanwhile, considering that E3 ubiquitin ligase has the problem of tissue-specific targeting, in the future, we need to prioritize the screening of E3 ubiquitin ligase that is highly expressed in the lungs as a target to regulate the treatment of IPF and using organoid models to dissect E3 functions in IPF niche. In summary Future research should aim to precisely identify and inhibit key drivers of pulmonary fibrogenesis, thereby providing new therapeutic targets and diagnostic strategies for pulmonary fibrosis.
